# Virus Adaptation by Manipulation of Host's Gene Expression

**DOI:** 10.1371/journal.pone.0002397

**Published:** 2008-06-11

**Authors:** Patricia Agudelo-Romero, Pablo Carbonell, Miguel A. Perez-Amador, Santiago F. Elena

**Affiliations:** Instituto de Biología Molecular y Celular de Plantas, Consejo Superior de Investigaciones Científicas-UPV, València, Spain; Karolinska Institutet, Sweden

## Abstract

Viruses adapt to their hosts by evading defense mechanisms and taking over cellular metabolism for their own benefit. Alterations in cell metabolism as well as side-effects of antiviral responses contribute to symptoms development and virulence. Sometimes, a virus may spill over from its usual host species into a novel one, where usually will fail to successfully infect and further transmit to new host. However, in some cases, the virus transmits and persists after fixing beneficial mutations that allow for a better exploitation of the new host. This situation would represent a case for a new emerging virus. Here we report results from an evolution experiment in which a plant virus was allowed to infect and evolve on a naïve host. After 17 serial passages, the viral genome has accumulated only five changes, three of which were non-synonymous. An amino acid substitution in the viral VPg protein was responsible for the appearance of symptoms, whereas one substitution in the viral P3 protein the epistatically contributed to exacerbate severity. DNA microarray analyses show that the evolved and ancestral viruses affect the global patterns of host gene expression in radically different ways. A major difference is that genes involved in stress and pathogen response are not activated upon infection with the evolved virus, suggesting that selection has favored viral strategies to escape from host defenses.

## Introduction

One of the first consequences of organisms' adaptation to new environments is the manipulation of resources [1]–[4]. In this sense, the interaction between intracellular parasites and their hosts represents a paradigm of resource manipulation. In general, a virulent relationship results in the alteration of many aspects of cellular metabolism and development, which are taken over in the parasite's own benefit [5]–[7]. Whether the relationship between a host and a parasite evolves towards a more or less virulent or benign situation depends on several genetic and ecological factors that may affect virus accumulation and transmission between hosts [5]. Of particular interest in the context of emerging infectious diseases is the characterization of changes in the pathogen's genome that are responsible for adaptation to a new host after spilling over from the original one and to understand how these changes may alter host's metabolic and regulatory interactions.

High-density DNA microarrays offer an unparalleled view of the transcriptional events that underlie the host response to pathogens, providing a quantitative description of the behavior of tens of thousands of genes. In recent years, microarrays have been widely used to analyze the alteration of gene expression in host cells after infection with both animal [e.g.], [ 8]–[13] and plant [e.g.], [ 14]–[18] viruses. However, a common drawback of these previous studies is that experiments were either done in cell cultures [8]–[13], which always represent an artificial and oversimplified environment, or using host-virus pairs whose previous evolutionary history of association is unknown and the degree of impact of abiotic environmental factor uncontrolled [14], [17]. Therefore, the relevance of these studies and, more importantly, their evolutionary implications for the problem of emergent infectious diseases, are rather limited. In the following, the results from an experiment simulating the emergence of a plant virus that crossed the species barrier and is horizontally spreading on a population of partially-susceptible hosts are reported. Evolutionary changes in viral genome and phenotypic properties and, more importantly, in the way it interacts with its host's transcriptome are the focus of the study.

The pathosystem *Tobacco etch potyvirus* (TEV)-*Arabidopsis thaliana* ecotype L*er* has been chosen for the present study. TEV genome is composed of a 9.5 kb positive polarity single-strand RNA that encodes a large ORF whose translation generates a polyprotein that is subsequently self-processed by virus-encoded proteases into 10 mature peptides [19], [20]. TEV has a moderately wide host range infecting around 149 species from 19 families [21], although most of its natural hosts belong to the family *Solanaceae*. In these plants TEV induces stunting and mottling, necrotic etching and malformation in leafs [21]. *A. thaliana* ecotypes vary in their susceptibility to TEV. Some ecotypes (e.g., C24 and L*er*) are fully susceptible [22], [23] whereas many other (e.g., Col-0 and Ws-2) do not allow for systemic movement but support replication and cell-to-cell spread in inoculated leafs [22], [23]. *Arabidopsis* is a member of the family *Brassicaceae*, which belongs to a different order than the *Solanaceae* within the class *Magnoliopsida* [24]. Therefore, adaptation of TEV to *A. thaliana* represents a jump in host species at the taxonomic level of orders.

## Results and Discussion

### TEV adaptation to *A. thaliana*: phenotypic changes

**Figure 1:**
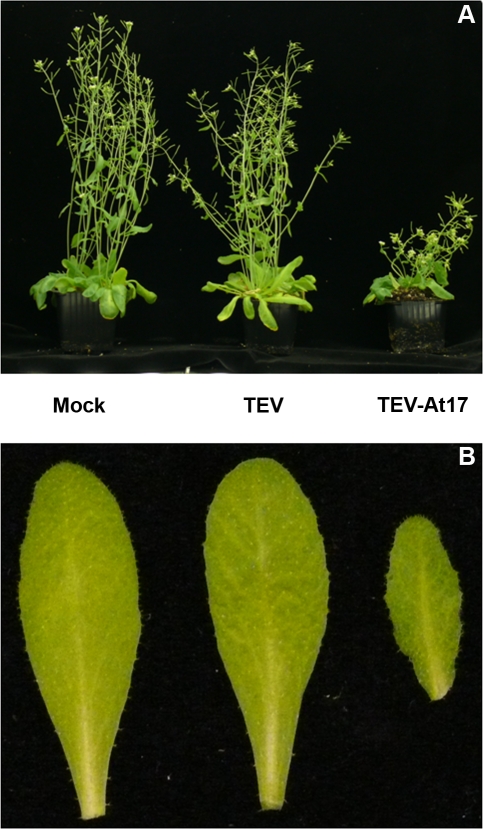
Symptoms developed 21 dpi by plants infected with ancestral and evolved TEV. (A) A mock-inoculated plant is shown at the left. Plants inoculated with the ancestral virus (TEV) show milder symptoms than plants inoculated with the evolved virus (TEV-At17). (B) Details of a healthy leaf from control plants (Mock), a leaf infected with the ancestral virus showing light vein clearing (TEV), and a leaf infected with the evolved virus (TEV-At17) and showing vein clearing and deformation.

The ancestral TEV was poorly adapted to *A. thaliana* L*er* and infection concurred with the development of very mild symptoms (Figure 1). Furthermore, 21 days post-inoculation (dpi), the viral load in infected plants, measured as the number of lesion-forming units (LFU) produced per milligram of tissue, was low, 48.33±2.95 LFU/mg (±SEM), and the infectivity of the newly produced viral particles (i.e., the efficiency of initiating a new infection using a normalized amount of viral particles) was as low as 17.95% [95% confidence interval (CI): 7.54–33.53%].

Viral particles obtained from a single tobacco plant were used to initiate an evolution experiment in *A. thaliana* L*er* plants. Seven independent lineages were founded. Each lineage consisted on 10 plants. Twenty-one dpi, positive infections were confirmed by Western blot hybridization using an anti-coat protein antibody (data not shown). One of the infected plants from each lineage was randomly chosen to be the source of viral particles for infecting the next batch of plants. This basic transfer protocol was serially repeated every three weeks. In six out of seven cases, lineages went to extinction as a consequence of decreases in viral loads beyond the threshold value that ensures efficient transmission. The only surviving lineage was maintained for 17 serial passages (hereafter TEV-At17). The viral load reached by TEV-At17 21 dpi, was 2138.38±134.08 LFU/mg. In other words, TEV-At17 accumulation was ∼44-fold larger than the value estimated for the ancestral TEV (two-sample *t*-test, *t*_43_ = 15.58, *P*<0.0001). Not only more viral particles were produced per gram of infected tissue, but also the infectivity of TEV-At17 was 100% (95% CI: 77.91–100%) and significantly larger than for the ancestral TEV (Binomial test, 1-tailed *P*<0.0001). Furthermore, symptoms induced by TEV-At17 were more severe (Figure 1), including stunting, vein clearing and leaf deformation.

### TEV adaptation to *A. thaliana*: genotypic changes

The above phenotypic changes have a correlate at the genetic level. Full-genome sequencing of TEV-At17 indicates that six changes have occurred during adaptation (first six rows in Table 1); three of them were non-synonymous. The first non-synonymous change, A1047V, affected the P3 protein. P3 localizes in nucleus and nucleoli in association with the NIa protein and participates in virus amplification through its interaction with the CI protein [20]. In other potyviruses, P3 is also involved in systemic movement [25], [26]. The second mutation is a T1210M replacement in the 6K1 peptide. This short peptide has been implicated in plant pathogenicity since its deletion results in symptomless infections [20]. Finally, the third amino acid replacement observed is L2013F in the VPg domain of the NIa protein. VPg is covalently attached to the 5′ end of the viral RNA and has essential functions in the viral replication and, relevant for the problem in hand, it has been reported as a key determinant in host-genotype specificity for systemic movement or replication [20] and it has been recently demonstrated that the proper interaction between the translation initiation factor eI4B and VPg is necessary for TEV infection [27]. In conclusion, these three mutations may explain the observed improvement in virus amplification and pathogenicity. The relevance of the three synonymous substitutions observed is not as clear, although their adaptive value cannot be ruled out.

**Table 1:** Symptoms associated to the five mutations identified in the evolved virus TEV-At17.

Nucleotide change	Protein and amino acid change	Symptoms severity
U537C	P1 synonymous	−
C3140U	P3 A1047V	−
C2518U	6K1 T1210M	−
C6037U	VPg L2013F	+
C6906U	NIa-Pro synonymous	−
	A1047V/T1210M	−
	A1047V/L2013F	+++
	T1210M/L2013F	+
	A1047V/T1210M/L2013F	+++

The three possible non-synonymous double mutants and the triple non-synonymous mutant were also constructed and their effect in symptoms development evaluated (Figure S1).

To further characterize the relationship between these changes and symptoms severity, we introduced them by site-directed mutagenesis in the ancestral TEV clone. In addition, all three possible pairs of non-synonymous mutations and the triple non-synonymous mutant were also created. *A. thaliana* L*er* plants were inoculated with these nine mutant clones and maintained in the same growth conditions for three weeks. The results of this experiment are summarized in Table 1. All mutant genotypes were viable and replicated and accumulated in the plants, as confirmed by Western blot analysis (data not shown). Among the three single mutants, the only clone that produced visible symptoms was the one containing the L2013F allele in VPg. These symptoms were, nonetheless, qualitatively milder than those produced by TEV-At17 (Figure S1). Concerning the three double mutants, only the combination of VPg and P3 substitutions induced symptoms that were qualitatively more severe than those produced by the single VPg L2013F mutant (Table 1) and almost as severe as those observed for TEV-At17. By contrast, mutation 6K1 T1210M does not have any effect on aggravating the symptoms associated with VPg L2013F. The combination of substitutions in P3 and 6K1 did not produce any symptom. Finally, the triple mutant recreated the strong symptoms characteristic of TEV-At17 (Table 1 and Figure S1). All together, these results suggest that the presence of substitution L2013F in the VPg protein is enough for triggering symptoms and that the severity of these symptoms is enhanced by the presence of substitution A1047V in P3, suggesting an epistatic interaction between these two mutations. The role of substitution T1210M in the 6K1 peptide remains unclear.

It has been recently reported that the correct interaction between potyvirus' VPg and host's eIF4E is required to initiate a successful infection [27]. Recessive resistance of peppers to potyvirus depends on the substitution of relevant amino acid residues in eIF4E that disrupt the normal interaction between this translation factor and VPg. Resistance-breaking viral strains restore the normal interaction [27]. Therefore, we can hypothesize that TEV-AT17 has enhanced its ability to infect *A. thaliana* L*er* by improving the interaction of its VPg with the host's translation initiation factor eIF4E.

### Differential effect of evolved and ancestral viruses on the overall pattern of host gene expression

**Figure 2:**
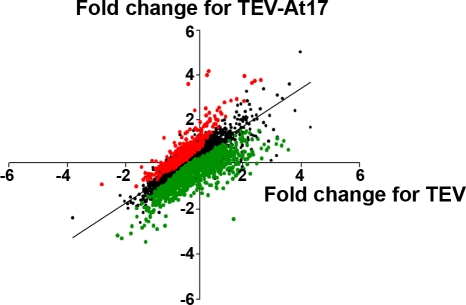
Scatter plot of expression patterns of 12,120 genes between TEV- versus TEV-At17-infected plants. Expression data were normalized by the median value obtained for the mock-inoculated plants. Green and red spots represent genes whose expression was significantly down- and up-regulated, respectively, in plants infected with TEV-At17 relative to those infected with the ancestral TEV virus. Black spots correspond to genes whose expression did not differentially respond to the infection of each viral genotype.

Next, we sought to unravel what component of the plant gene interaction networks and metabolic pathways have been targeted by the virus during its adaptation to *A. thaliana* L*er*. Our goal is not to identify single genes but rather global transcriptomic changes. Long-oligonucleotide microarrays representing almost all genes in *A. thaliana* genome have been used to this end. Five replicates were analyzed per experimental treatment (control mock-inoculated plants, and plants infected with TEV and TEV-At17) using a global reference experimental design. After quality analysis, a total of 13,722 spots, corresponding to 12,180 genes, were considered as valid for further analyses (Table S1). Data were normalized to the median expression of non-infected plants, and thus they reflect biological differences in gene expression in each sample analyzed. Statistical analysis allowed identification of genes whose expression responded differentially upon infection with either TEV or TEV-At17 (Figure 2). When comparing global patterns of gene expression in plants infected with ancestral and adapted viruses, 496 genes showed higher expression and 1,322 genes lower expression in TEV-At17 infections than in TEV infections (Figure 2 and Tables S2 and S3); which represents 2.7 times more down-regulated than up-regulated genes (Binomial test, *P*<0.0001).

**Figure 3:**
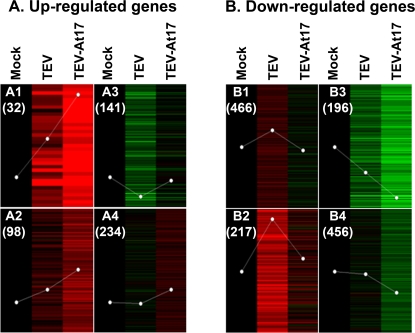
Self-organization maps (SOMs) showing different patterns of gene expression. Gene expression patterns for control (Mock), TEV-infected and TEV-At17-infected plants are organized into SOMs (labeled as 1 to 4 on panels A and B). The actual number of genes belonging to each SOM category is indicated below the corresponding label (in parenthesis). Green ranges are used to represent different levels of down-regulation relative to the control uninfected plants; red ranges are used to represent different magnitudes of up-regulation relative to uninfected plants. The brighter the color, the larger the difference in gene expression. (A) Plant genes whose expression is up-regulated upon infection with TEV-At17 compared with plants infected with the ancestral TEV. (B) Genes whose expression is down-regulated in plants infected with TEV-At17 compared with plants infected with the ancestral TEV.

Differentially expressed genes were grouped according to self-organizing maps (SOM) (Figure 3 and Table S4). Three global patterns of gene expression were observed among genes that were up-regulated by TEV-At17 infection (Figure 3A). The first pattern (SOMs A1 plus A2) corresponds to 130 genes whose expression was activated by both viruses but the magnitude of expression was magnified by TEV-At17. Genes belonging to this category include the pathogenesis-related protein *PR1*, which is well known to be a marker for the activation of salicylic acid-dependent defenses, such as the systemic acquired resistance (SAR) pathway [28], [29]. The second pattern (SOM A3) corresponds with 141 genes that were down-regulated after infection with TEV but showed expression levels similar to uninfected plants when infected with TEV-At17. The third pattern (SOM A4) corresponds to 234 genes whose expression was not significantly affected by TEV infection but show increased expression after infection with TEV-At17.

Three distinct patterns were also observed among genes down-regulated after infection with TEV-At17 relative to the infection with TEV (Figure 3B). The first pattern (SOMs B1 plus B2) represents 683 genes that were over-expressed by plants infected with TEV but infection with TEV-At17 resulted in expression levels similar to those observed in uninfected plants. Interestingly, proteins related with disease response such as *PR5* and several other *PR*-like proteins as well as four proteins of the TIR-NBS-LRR class [29], [30] belong to this category. The second pattern (SOM B3) includes 196 genes that were down-regulated after infection with both ancestral and evolved viruses, although the magnitude of down-regulation was larger for TEV-At17. Finally, the third pattern (SOM B4) corresponds to 456 genes whose expression was not affected by TEV but showed lower expression when TEV-At17 infected the plants.

The expression of transcription factors (TF) was also differentially affected by TEV and TEV-At17. Table S5 shows the list of differentially up- and down-regulated TF in plants infected with each type of virus. Fifty-one TFs, belonging to 20 families, were up-regulated whereas 84 TFs, from 27 families, were down-regulated, including 13 ethylene-responsive binding factors (ERF), after infection with TEV-At. ERFs are linked to stress responses [31] and delays in ERF induction had been described in *A. thaliana* plants infected with virulent strains of the bacteria *Pseudomonas syringae* when compared with avirulent strains of the same bacteria [31].

### Viral adaptation by avoidance of plant defenses

Next, we examined the distribution of genes involved in related biological processes that are differentially affected by TEV and TEV-At17 (i.e., gene ontologies (GO) categories [32]). The algorithm implemented in FatiGO [33] was applied to the non-redundant gene list grouped in each SOM (results are shown in Table S6). Only a significant differential category, response to salt stress, was identified for the SOM A3 of up-regulated genes shown in Figure 3A. By contrast, a large number of GO terms show significant over- and under-representation in the differentially down-regulated genes (Figure 3B). Table 2 shows the non-redundant functional categories that correspond to SOMs B1 plus B2 (i.e., genes over-expressed after infection by TEV but not differing from uninfected plants when infected with TEV-At17). Interestingly, significantly over-represented genes belong to functional categories which are related to plant responses to different abiotic (wounding, light intensity, temperature, salinity) and biotic stresses. Furthermore, genes involved in the SAR and in the activation of innate immune responses [29] were not expressed on plants infected with TEV-At17 while they were over-expressed on plants infected with the ancestral TEV, suggesting that the evolved virus acquired the ability to evade certain plant defense mechanisms, perhaps explaining the observed increase of viral load. Genes involved in basic cellular processes such as nucleic acid metabolism, translation and proteolysis were under-represented among down-regulated genes in SOMs B1 plus B2 (Table 2), suggesting that the plant may be compensating for the consumption of these resources by an increased viral replication.

**Table 2:** Non-redundant GO categories differentially represented in SOMs B1 plus B2 of down-regulated genes

GO term	GO level	Differentially expressed (%)	Total genes in the class (%)	*P*
**Over represented**				
Response to wounding	4	4.26	0.76	<0.001
Response to hormone stimulus	4	9.09	4.85	0.048
Cell-to-cell signaling	4	1.42	0.19	0.050
Response to cold	5	4.82	1.43	0.008
Response to bacterium	5	3.54	0.82	0.009
Thigmotropism	5	0.64	0.00	0.048
Hyperosmotic salinity response	6	2.47	0.27	0.010
Protein modification process	6	24.69	15.02	0.010
Response to light intensity	6	2.06	0.27	0.047
Protein amino acid phosphorylation	7	26.56	14.38	0.002
MAPKKK cascade	7	1.56	0.07	0.047
Systemic acquired resistance	8	5.38	0.47	0.013
Activation of innate immune resistance	9	10.53	0.61	0.015
**Under represented**				
Nucleobase, nucleoside, nucleotide, and nucleic acid metabolic processes	4	13.64	22.54	0.004
Regulation of cellular processes	4	9.94	16.22	0.048
Proteolysis	6	2.06	7.98	0.011

A single significant GO category was also found in SOM B3 of down-regulated genes (Figure 3B), that is, gene expression was repressed in presence of both viruses but to a larger extent when TEV-At17 was infecting plants. Genes involved in response to auxin were under-expressed to a larger extent by plants infected with TEV-At17 than with TEV.

### Concluding remarks

We have shown that adaptation of a virus to a new host occurs by few changes in viral genome. The increase in viral fitness correlates with deep changes in the patterns of host's gene expression, illustrating that the subtle but dynamic interplay between the pathogen and the plant shifts as the virus adapts to its host. Under the experimental conditions imposed, it may be speculated that natural selection may had favored viral genomes that avoided plant defense mechanisms as suggested by the observation of stress-related genes not being activated after infection with the evolved virus (Table 2). Therefore, perhaps as a consequence, increases in the strength of symptoms, virus accumulation and transmissibility have been observed. These phenotypic changes are associated to a few genomic changes fixed in the viral genome. In particular, the development of symptoms is associated to a single substitution in the viral VPg protein, whereas ulterior mutations in other viral components simply magnify symptoms. Our starting hypothesis was that viral adaptation occurs throughout the integration of viral replication processes within host physiology and circuitry of genetic and metabolic interactions. Necessarily, this integration has to affect the patterns of host's gene expression. Our experiments directly test this hypothesis, supporting its validity and, furthermore, pinpointing some physiological processes that may be targeted by the virus as it improves its fitness. The obvious follow-up of this study is to dissect the physiological processes and identify, whenever possible, the precise steps and proteins that are getting targeted by the virus during its adaptation.

Serial-passage experiments simulating horizontal transmission are well known to produce increases on parasite's virulence due to enhanced within-host competition among pathogenic strains, the decoupling between intra-host growth rate and transmission rate, and the lack of evolutionary innovation in the host [34]. The outcome of a different experimental design in which transmission would be vertical, and hence making high virulence detrimental, or in which virus and host are engaged in a coevolutionary arms-race may produce different results; perhaps with the evolution of a less severe virus and different alterations in plant gene expression.

Finally, the findings here reported call for extra precaution when analyzing data from microarray experiments seeking for the effect of pathogen's infection on host gene expression: the pathogen effect on host's transcriptomic profiles would depend on the degree of adaptation of the pathogen to the host and to environmental conditions. Therefore, the only fully meaningful studies would be those in which pathogens and their experimental hosts would have an evolutionary history of association in the experimental growth conditions, whereas results from studies in which hosts are infected with naïve pathogens or the effect of environmental variables on pathogen's growth remain uncontrolled would be of very limited interest.

## Materials and Methods

### Virus and plants

An infectious clone pTEV-7DA [35] (GeneBank DQ986288), kindly provided by Prof. J.C. Carrington (Oregon State Univ.) was used as ancestor virus. This infectious clone contains a full-length cDNA of TEV and a 44 nt long poly-T tail followed by a *Bgl*II restriction site cloned into the pGEM-4 vector downstream of the SP6 promoter. 5′ capped infectious RNA was obtained upon transcription of *Bgl*II-digested pTEV-7DA using SP6 mMESSAGE mMACHINE kit (Ambion). A stock of ancestral TEV viral particles was generated as follows. Five µg of RNA transcripts were rub-inoculated into the third true leaf of four-week old *Nicotiana tabacum* var Xanthi plants. Afterwards, plants were maintained in the green house at 25°C and 16 h light photoperiod. Seven dpi, virions were purified as described elsewhere [36], aliquoted and stored at −80°C.

The viral load reached by replicating TEV populations in *A. thaliana* was estimated by the dilution-inoculation assay method on the local-lesion host *Chenopodium quinoa* [37]. In short, 2 g of tissue was ground in 1 mL of 0.5 M phosphate buffer. Four different leafs from each one of three different 4-week-old *C. quinoa* plants were rub-inoculated with 5 µL of undiluted, 5- and 10-fold diluted virus, respectively; 100 mg/mL carborundum were added to facilitate inoculation. Nine dpi, the number of local lesions was recorded and transformed into viral infectious loads (LFU/mg) by estimating the intercept of the regression line of the observed number of lesions on the dilution factor.

*A. thaliana* L*er* seeds were obtained from Lehle Seeds (cat. # WT-04 18 01).

### Experimental evolution protocol

Seven independent evolution lineages of TEV were maintained by serial passages until extinction or up to 17 passages. All evolving lineages were initiated from the ancestral TEV stock population. Therefore, initial viral genetic variation among inoculated *A. thaliana* plants was minimal. To maximize transmission success, 10 plants were inoculated per lineage. Plants were inoculated between growth stages 3.5 and 3.7 [38]. Plants were maintained at 25°C and 16 h light photoperiod. Successful infections were confirmed by Western blot hybridization analysis 21 dpi using commercial antibodies anti-coat protein conjugated with horseradish peroxidase (Agdia). One gram of leaf tissue from a randomly-chosen infected plant per lineage were carefully ground in 1 mL 0.5 M phosphate buffer (pH = 8.0) and used to inoculate the next batch of 10 plants. Plants were always inoculated with similar viral doses.

### Genome sequencing

The consensus full-genome sequence of TEV-At17 was obtained following standard methods. In short, RNA was extracted using the RNeasy® Plant Mini kit (Quiagen), it was reverse-transcribed using MMuLV polymerase (Fermentas) and PCR amplified with *Taq* polymerase (Roche). The ABI Prism Big Dye Terminator Cycle Sequencing Kit 3.1 (Applied Biosystems) was used for cycle sequencing with fluorescently labeled dideoxynucleotides. Cycle sequencing reactions were carried out on a GeneAmp PCR System 9700 thermal cycler (Applied Biosystems). Labeled products were resolved in an ABI 3100 Genetic Analyzer (Applied Biosystems). Seven pairs of specific primers were used to amplify the 9.5 kb of TEV genome. The resulting fragments were overlapping, facilitating the task of fragment sequence assembly. Sequences were processed and analyzed with the STADEN 1.4b1. The 5′- and 3′-ends were sequenced by the RACE-PCR method [39].

### Site-directed mutagenesis

The seven mutant genotypes created in this study were generated by site-directed mutagenesis using the Quikchange® II XL kit (Stratagene) and following the indications of the manufacturer. Mutagenic primers were also designed according to Stratagene recommendations. To minimize unwanted errors during the mutagenesis process, the kit incorporates the *PfuUltra*™ high fidelity DNA polymerase. The presence of the desired mutation was confirmed by sequencing. To assess the presence of undesired mutations on each clone, the Surveyor™ Mutation Detection Kit Standard Gel Electrophoresis (Transgenomic) was employed. All six mutant genotypes presented the expected genome-wide band pattern.

### RNA extraction and microarray hybridization

Total RNA was extracted from control and infected plants and used in an amplification reaction with the MessageAmp II aRNA Amplification kit (Ambion) following manufacturer's instructions.

Five replicates for each sample category were generated, and compared with a global reference, generated from an equimolar mix of amplified RNAs from each of the 15 plants. RNA from each individual sample, plus the reference, were amplified, and used for labeling. For each category, three samples were labeled with Cy5 and two with Cy3, and compared with the corresponding reversed-labeled reference mix. Long 70-mers oligonucleotide microarrays, provided by Dr. D. Galbraith (Univ. Arizona), contain 29,110 probes from the Qiagen-Operon *Arabidopsis* Genome Array Ready Oligo Set (AROS) Version 3.0. This oligo set represents 26,173 protein-coding genes, 28,964 protein-coding gene transcripts and 87 miRNAs and is based on the ATH1 release 5.0 of the TIGR *Arabidopsis* genome annotation database (www.tigr.org/tdb/e2k1/ath1/) and release 4.0 of the miRNA Registry at the Sanger Institute (www.sanger.ac.uk/Software/Rfam/mirna/index.shtml). Further information can be found at the Operon website (omad.operon.com/download/index.php). Oligos were rehydrated and immobilized by UV irradiation. Slides were then washed twice in 0.1% SDS, 4 times in water, dipped in 96% ethanol for 1 min, and dried by centrifugation. Slides were prehybridized 30 min at 42°C with 100 µL of 6× SSC, 1% BSA and 0.5% SDS, under a 60×22 mm coverslip LifterSlip (Erie Scientific) in an ArrayIt microarray hybridization cassette (TeleChem). Slides were then rinsed five times in H_2_O and dried by centrifugation. Slides were hybridized immediately. Labeled RNA was used to hybridize the slides basically as described in [40]. After hybridization and wash, slides were scanned at 532 nm for the Cy3 and 635 nm for the Cy5 dyes, with a GenePix 4000B scanner (Axon Molecular Devices), at 10 nm resolution and 100% laser power. Photomultiplier tube voltages were adjusted to equal the overall signal intensity for each channel, to increase signal-to-noise ratio, and to reduce number of spots with saturated pixels. Spot intensities were quantified using GenePix Pro 6.0 (Axon Molecular Devices).

Microarray raw data were deposited in the NCBI's GEO database under accession GSE11088.

### Microarray data analysis

Spots with a net intensity in both channels lower than the median signal background plus twice standard deviations were removed as low signal spots. Data were normalized by median global intensity and with LOWESS correction [41] using the Acuity 4.0 software (Axon Molecular Devices). Finally, only probes for which a valid data was obtained in at least 13 out of the 15 slides were considered for further analysis (13,722 spots; Table S1). Median, mean and standard deviations were calculated from each treatment (control, TEV- and TEV-At17-infected plants), and all data were normalized to the median of the expression in control samples. To detect differentially expressed genes in plants infected with TEV-At17 compared to TEV, data were analyzed with the SAM package [42], using two-class comparison (TEV versus TEV-At17) with a false discovery rate (FDR) of 5.38% with no fold-change cut-off. Differentially over- and under-expressed genes were grouped in 2×2 self-organizing maps (SOMs) [43] using Acuity with Euclidean squared similarity metrics. Gene lists were further analyzed with FatiGO [33] to find differential distributions of gene ontology (GO) terms between statistically differential genes in each SOM and the rest of genes in the microarray, with *P* values adjusted after correcting for multiple testing [33]. SAM analysis at 1% FDR gave qualitatively identical results, confirming their robustness to changes in arbitrarily-chosen statistical thresholds.

## Supporting Information

Figure S1 Representative plants showing the symptoms induced by several of the viral genotypes described in Table 1.
(2.50 MB TIF)

Table S1 Gene expression data from DNA microarray analysis. Mock, control non-infected plants; TEV, plants infected with the ancestral virus; TEV-At17, plants infected with the evolved virus. A total of 13,722 spots were considered to give high quality expression data. Median, mean and standard deviation were calculated for each group of samples and all data were normalized by the median expression in the control plants. Gene name and annotation are included.
(9.36 MB XLS)

Table S2 Significantly up-regulated genes between TEV- and TEV-At17-infected plants. Genes were ordered based on the score in SAM output with a FDR of 5.38% (533 spots, corresponding with 496 genes).
(0.49 MB XLS)

Table S3 Significantly down-regulated genes between TEV- and TEV-At17-infected plants. Genes were ordered based on the score in SAM output with a FDR of 5.38% (1378 spots, corresponding with 1322 genes).
(1.12 MB XLS)

Table S4 SOM clustering of significant genes, both up- and down-regulated, between TEV- and TEV-At17-infected plants. Genes belonging to each of the eight SOMs in Figure 3 are listed on different spreadsheets, along with their annotation and mean expression data in control and in TEV- and TEV-At17-infected plants.
(0.48 MB XLS)

Table S5 Transcription factors differentially expressed after infection with TEV and TEV-At17. A. thaliana transcription factors and other transcription regulators were mainly downloaded from arabidopsis.med.ohio-state.edu/AtTFDB/index.jsp, and collapsed with the significantly up-regulated (Table S2) and down-regulated (Table S3) genes between TEV- and TEV-At17-infected plants, to generate a list of differentially expressed transcription factors. Mean and standard deviations are indicated for control, TEV- and TEV-At17-infected plants.
(0.08 MB XLS)

Table S6 Differential GO categories among differential genes grouped by SOM. FatiGO analysis was carried out for each SOM in Figure 3. Differential categories were identified for down-regulated genes in SOMs B1 plus B2 and B3 (Figure 3B) and in up-regulated genes in SOM A3 (Figure 3A). List1 includes the differential genes (gene name, number and percentage) belonging to each GO category, while List2 include the rest of genes in the same GO category represented in the microarray. Unadjusted and adjusted P values after correcting for multiple-tests are also indicated.
(0.07 MB XLS)
