# Supplementary figures and images for: Virus Adaptation by Manipulation of Host's Gene Expression
Source: PLoS One. 2008 Jun 11;3(6):e2397. doi: 10.1371/journal.pone.0002397 (PMC2398778; doi:10.1371/journal.pone.0002397)

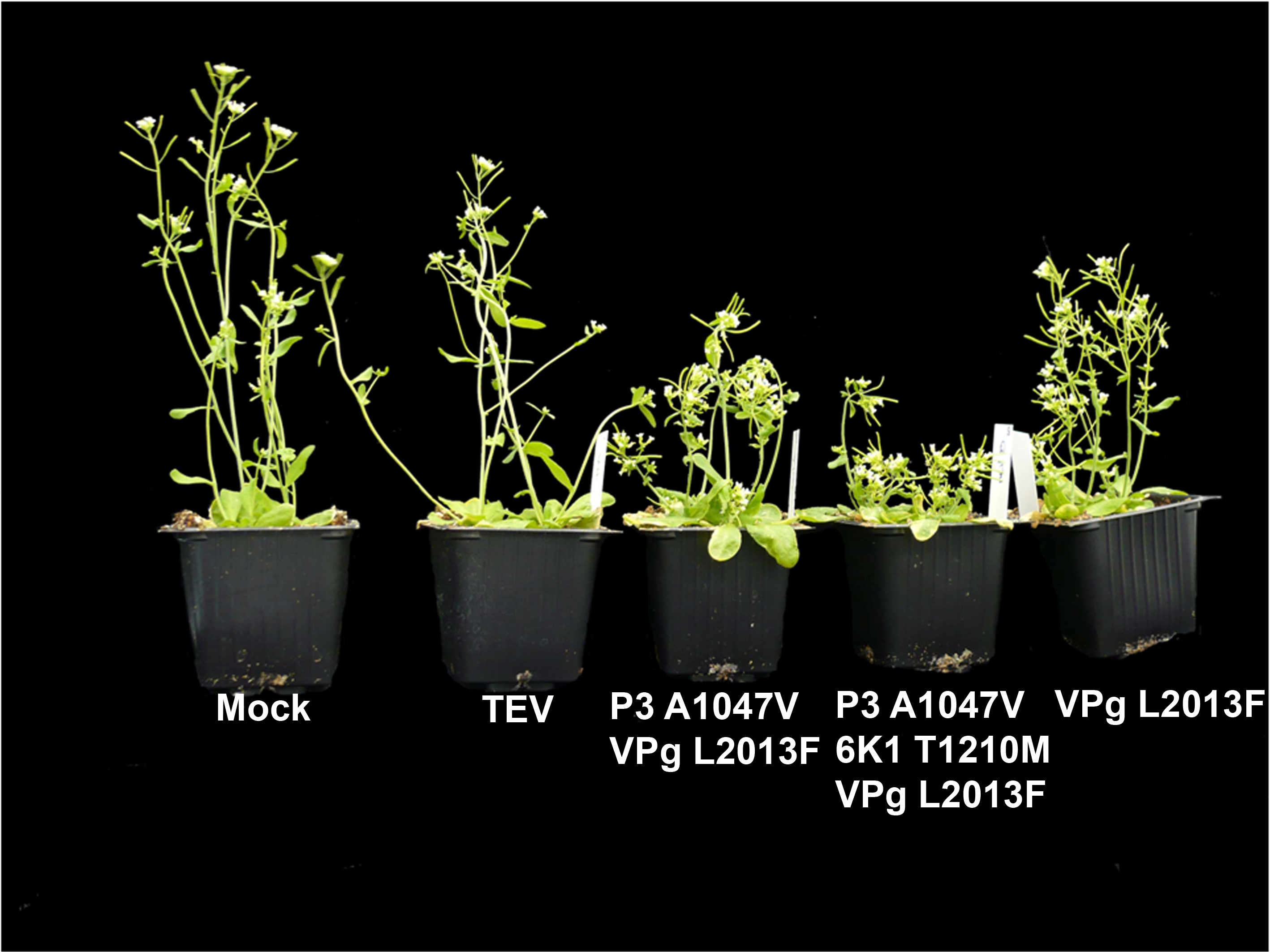

Supplement: Figure S1 — Representative plants showing the symptoms induced by several of the viral genotypes described in Table 1. (2.50 MB TIF) [file pone.0002397.s001.tif]
